# Magnetic droplet nucleation boundary in orthogonal spin-torque nano-oscillators

**DOI:** 10.1038/ncomms11209

**Published:** 2016-04-18

**Authors:** Sunjae Chung, Anders Eklund, Ezio Iacocca, Seyed Majid Mohseni, Sohrab R. Sani, Lake Bookman, Mark A. Hoefer, Randy K. Dumas, Johan Åkerman

**Affiliations:** 1 Department of Physics, University of Gothenburg, 412 96 Gothenburg, Sweden; 2 Materials and Nano Physics, School of ICT, KTH-Royal Institute of Technology, Electrum 229, 164 40 Kista, Sweden; 3 Integrated Devices and Circuits, School of ICT, KTH-Royal Institute of Technology, Electrum, 229, 164 40 Kista, Sweden; 4 Department of Applied Mathematics, University of Colorado, Boulder, Colorado 80309-0526, USA; 5 Department of Physics, Division for condensed matter theory, Chalmers University of Technology, Gothenburg 412 96, Sweden; 6 Department of Physics, Shahid Beheshti University, Tehran 19839, Iran; 7 Department of Mathematics, Yale University, New Haven, Connecticut 06511, USA

## Abstract

Static and dynamic magnetic solitons play a critical role in applied nanomagnetism. Magnetic droplets, a type of non-topological dissipative soliton, can be nucleated and sustained in nanocontact spin-torque oscillators with perpendicular magnetic anisotropy free layers. Here, we perform a detailed experimental determination of the full droplet nucleation boundary in the current–field plane for a wide range of nanocontact sizes and demonstrate its excellent agreement with an analytical expression originating from a stability analysis. Our results reconcile recent contradicting reports of the field dependence of the droplet nucleation. Furthermore, our analytical model both highlights the relation between the fixed layer material and the droplet nucleation current magnitude, and provides an accurate method to experimentally determine the spin transfer torque asymmetry of each device.

## Abstract (web summary)

Magnetic droplets occur in nanocontact spin-torque oscillators with perpendicular anisotropy, forming part of a family of particle-like magnetic objects, which may be excited for high-frequency applications. Here, the authors determine a current–field phase diagram for magnetic droplet nucleation.

## Graphical abstract

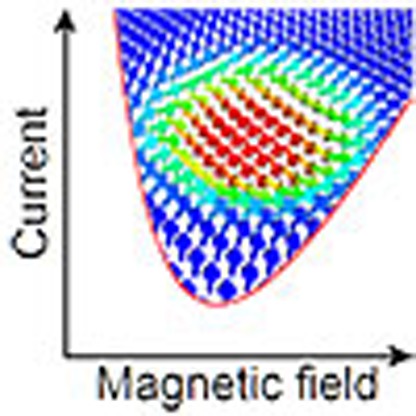

Magnetic solitons^1^ play a critically important role in nanomagnetism with the one-dimensional domain wall being the most fundamental, defining the typical scale for magnetic textures and enabling high-density magnetic storage. Two-dimensional solitons, such as vortices^2^,^3^, skyrmions^4^,^5^,^6^,^7^ and dynamical skyrmions^8^ have also received significant attention for both memory and oscillator applications^8^,^9^,^10^. Common to these solitons is their nonzero topological charge, which affords them particular stability in extended films. In addition to such topological solitons, there also exists a family of nontopological and intrinsically dynamical solitons, of which the two most recent experimental examples are the spin wave bullet^11^,^12^,^13^,^14^,^15^,^16^ and the magnetic droplet^17^,^18^,^19^,^20^,^21^,^22^,^23^,^24^,^25^, with very high amplitudes of self-localized spin waves at frequencies relevant for microwave applications.

Magnetic droplets (droplets from here on) were suggested in 2010 (ref. 17) as a dissipative analogue of conservative magnon drops introduced in the 1970s^26^,^27^. Magnon drops can exist in magnetic thin films with perpendicular magnetic anisotropy (PMA), where the PMA leads to an attractive force between the magnons. In the absence of damping, magnons can condense leading to a partial magnetization reversal and a precessing boundary with an envelope given by a balance between anisotropy and exchange. Although real magnetic materials always exhibit non-zero damping, spin transfer torque (STT)^28^,^29^ can locally cancel the damping in devices known as nanocontact spin-torque oscillators (NC-STO)^22^,^30^,^31^,^32^. Following the first NC-STOs with PMA free layers^33^,^34^, droplets were finally demonstrated^19^ in 2013.

Although droplet theory^17^ has predominantly been applied to all-perpendicular spin valves, where both the free and fixed layer have strong PMA, experiments^19^,^20^,^21^,^22^,^23^,^24^,^25^ have used NC-STOs with in-plane fixed layers, for which the current–field phase diagram of the droplet qualitatively differs from the theoretical all-perpendicular case. Although experiments and theory both show a linear field dependence of the droplet nucleation current at high perpendicular fields, where the fixed layer is fully saturated out of plane^23^, the droplet nucleation current at low fields instead scales well with the perpendicular component of the spin polarization^19^, and acquires an inverse applied field (1/*H*) dependence. However, at intermediate fields, both these descriptions break down^21^.

In the following sections, we study these opposing trends by first mapping out the complete and detailed current–field phase diagram for droplet nucleation in a wide range of NC size, and then deriving a theoretical model that includes both the applied field, and the field-dependent perpendicular component of the fixed-layer spin polarization. We find an excellent agreement between model and experiment, including the existence of a minimum droplet nucleation current at intermediate fields.

## Results

### Orthogonal NC-STO characterization

**Figure 1:**
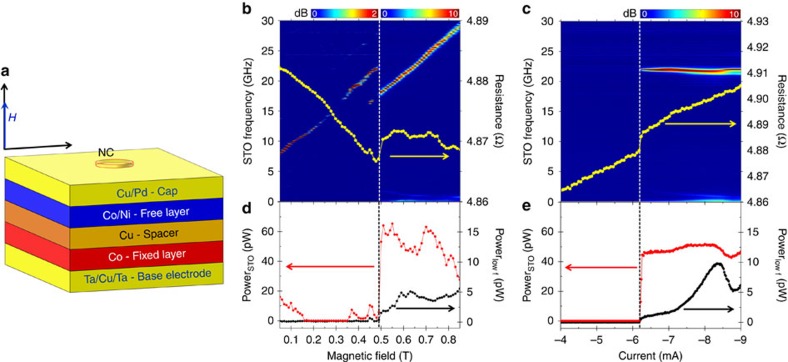
Orthogonal NC-STO characterization. (**a**) Schematic of the orthogonal NC-STOs showing a pseudo-spin-valve composed of a Co fixed layer, a Cu spacer and a Co/Ni free layer with perpendicular magnetic anisotropy. The current enters the stack through a nanocontact (NC). The field *H* is applied perpendicularly to the plane. (**b**,**c**) Representative power density spectra as a function of field magnitude and current are shown in **b** and **c**, respectively. Droplet nucleation is observed as a frequency drop accompanied by a dramatic increase in power. The nucleation can also be monitored by a jump in resistance, as shown by the yellow data in **b** and **c**. (**d**,**e**) Show corresponding microwave power integrated around the main high-frequency peak (red) as well as in a low-frequency region from 0.1 to 1 GHz (black). Both the main signal power and the onset of low-frequency dynamics are clear signatures of droplet nucleation.

The investigated devices are orthogonal NC-STOs (Fig. 1a) with a perpendicularly magnetized Co/Ni free layer, and an in-plane magnetized Co fixed layer (see ‘Methods' section). The Co/Ni free layer has a saturation magnetization *M*_s_ of *μ*_0_*M*_s_=0.9 T and a PMA field of *μ*_0_*H*_k_=1.35 T, where *μ*_0_ is the permeability of free space; the Co fixed layer has a saturation magnetization of about *μ*_0_*M*_s,p_=1.6 T. Figure 1b shows the field-dependent NC-STO magnetodynamics for a NC radius of 40 nm, where the perpendicular field *μ*_0_*H* was swept from 0.05 to 0.85 T at a constant device current of *I*_dc_=−7.7 mA, where the negative current polarity indicates electrons flowing from the free to the fixed layer. For low magnetic fields, a faint signal is observed, corresponding to moderate-angle precession close to the ferromagnetic resonance frequency^33^,^34^. Above a critical field of about 0.48 T, a sudden frequency drop and a dramatic increase in microwave power (red data points in Fig. 1d) are observed, marking the nucleation of a droplet. A similar droplet nucleation transition is observed in Fig. 1c,e as a function of current in a constant perpendicular field of *μ*_0_*H*=0.625 T. The yellow line in Fig. 1b,c shows how the NC-STO magnetoresistance (MR) exhibits a step-like increase at the droplet nucleation stemming from the partial reversal of the free layer magnetization underneath the NC. The nucleation transition can also be detected by measuring the integrated microwave noise power between 0.1 and 1 GHz, black data points in Fig. 1d,e. This technique is based on the fact that broad, low-frequency features in the spectrum can be related to random transitions between different dynamical regimes. In our experiments, we hypothesize that only thermally driven spin waves and droplets are supported (corroborated by the theory given in ref. 17), suggesting that the appearance of low-frequency noise is related to the nucleation and drift instabilities of droplets. This conclusion is supported by the good agreement between the MR and microwave noise power techniques to detect a droplet, as shown in Fig. 1d,e. These are particularly useful at high fields, where the excited microwave frequencies surpass the bandwidth of our low noise amplifier (30 GHz) and spectrum analyzer (40 GHz).

### Magnetoresistance measurement

**Figure 2:**
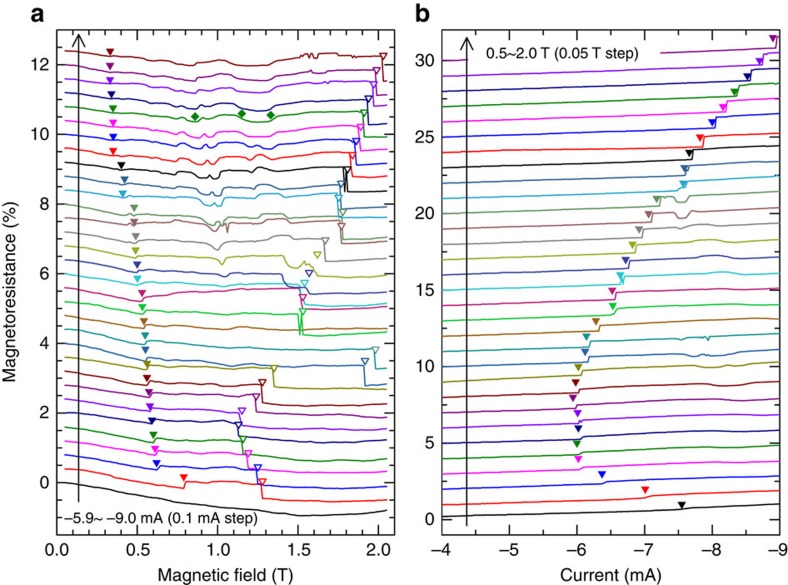
Magnetoresistance measurements. (**a**,**b**) Show the magnetoresistance (MR) measured by sweeping either the field or current, respectively. Each MR curve is vertically shifted for clarity. The transition resistance for droplet nucleation (collapse) is shown by a solid (empty) triangle. The solid diamonds in **a** indicate minor MR variations after the droplet has been nucleated and can be attributed to unstable dynamics.

Detailed MR measurements for an NC with a radius of 40 nm are plotted in Fig. 2 as a function of both field and current. Figure 2a shows field-dependent sweeps from 0.05 to 2.05 T using a field step of 0.01 T. In between each field-sweep, the current is varied in steps of −0.1 mA from −5.9 to −9 mA with data from each sweep vertically offset for clarity. Filled triangles indicate droplet nucleation, whereas hollow triangles indicate droplet collapse. In addition, small MR fluctuations are observed inside the region where the droplet exists (diamond markers in Fig. 2). These minor features, while not further analysed, may be attributable to small changes in the droplet dynamics generated by inhomogeneities in the magnetic films^23^. Although the droplet nucleation field shows a monotonic dependence on current, the droplet collapse field sometimes displays a much greater variation, for example, at −6.9 and −7.0 mA and at the lowest currents. We attribute this variation to a general high degree of drift instability, except at a few current conditions where the droplet appears more stable. The high microwave noise power observed at most field and current conditions where the droplet exists corroborates this picture and suggests that the droplet leaves the NC region relatively quickly after nucleation to give way for the immediate renucleation of another droplet, in good agreement with numerical predictions^17^. At field and current conditions outside of the nucleation boundary, droplet renucleation is however no longer possible, and as a consequence, most of the collapse fields in Fig. 2a can be used to trace out the high field part of the nucleation boundary.

Figure 2b shows the corresponding current-dependent sweeps using a step of −0.02 mA, while the field is increased between each sweep from 0.5 to 2.0 T in steps of 0.05 T. The obtained MR data are again plotted with a vertical offset for clarity. For low field magnitudes, where MR steps are not as easily observed, we also rely on the spectral features (abrupt frequency drop, strong power increase, low-frequency microwave noise; see Fig. 1) to uniquely define nucleation. The field dependence of the nucleation current is clearly non-monotonic exhibiting a broad minimum at intermediate fields of about 0.8 T.

### Magnetic droplet nucleation boundary

**Figure 3:**
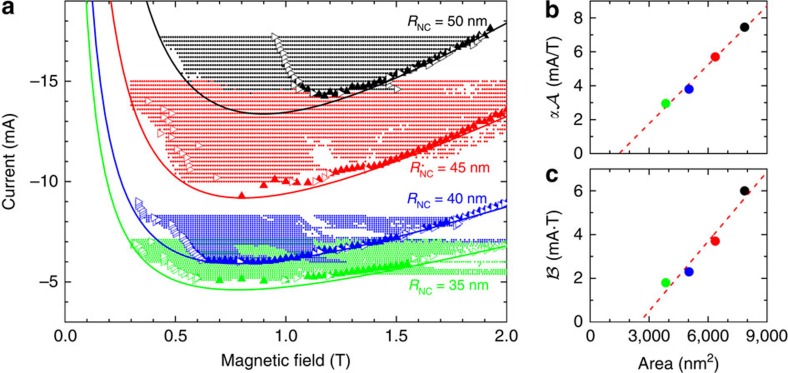
Droplet nucleation boundary. (**a**) Nucleation boundary found from the field sweeps (empty triangles), current sweeps (solid triangles) and low-frequency signals (solid circles) for devices with different NC radii *R*_NC_. Fits using equation (1) are shown by solid lines using the same colour code for each *R*_NC_. (**b**,**c**) The coefficients 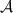 and 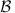 are shown in **b** and **c**, respectively, as a function of NC area utilizing the same colour code as shown in **a**. Linear fits in **b** and **c**, shown by red dashed lines, are used to calculate the spin-torque asymmetry and efficiency.

All nucleation and collapse points extracted from Fig. 2a,b can now be plotted in Fig. 3a together with the same analysis carried out on three additional NCs with nominal radii ranging from 35 to 50 nm. Clearly, the field-dependent scans (hollow triangles pointing sideways) and current-dependent scans (solid triangles pointing up) complement each other smoothly, justifying the uniqueness of the droplet nucleation conditions. Two trends can be noticed: at low fields, the nucleation current decreases as a function of field, which is consistent with previous reports^19^ in which the nucleation current was shown to be dominated by the perpendicular component of spin-polarized current, obeying a 1/*H* dependence; at high fields, the dependence is linear—in strong contrast to the predicted 1/*H* dependence^19^ but in good agreement with recent results^23^. A similar behaviour is obtained from current-sweep measurements. At intermediate fields, both trends are smoothly connected, suggesting that both regimes originate from the physical characteristics of the orthogonal NC-STO. In Fig. 3a, we have also added shaded areas corresponding to regions where the microwave noise power is higher than 0.3 pW as an additional indication of the presence of a droplet. It is interesting to note that for the largest NC, there is a wide region where the microwave noise, but not the resistance, indicates the presence of a droplet. As the resistance is a time-averaged measurement, this apparent discrepancy indicates that, for most of the time, there is no droplet present. In other words, the droplet is highly unstable and the time it takes for the droplet to drift away from the NC is shorter than the re-nucleation time. The greater drift instability is likely a consequence of the larger Oersted field and the higher local temperature, both of which are due to the much higher nucleation current necessary for droplet nucleation in the largest NC.

The droplet nucleation conditions observed in Fig. 3 theoretically coincide with the STT-driven spin wave instability threshold, given that the necessary conditions for droplet existence are fulfilled^17^, in particular, when a balance between PMA and exchange is reached^26^. In orthogonal NC-STOs, a perpendicular field along the PMA direction tilts the fixed layer orientation by an angle *θ*, defined with respect to the plane normal, directly given by a hard axis reversal condition cos(*θ*)=*H*/*M*_s,p_, applicable as long as *H*≤*M*_s,p_. By introducing this condition into the nucleation current equation, it can be shown that a 1/*H* dependence arises (see ref. 19 and the ‘Methods' section). This dependence is also expected in our experiments for fields below the fixed layer saturation *μ*_0_*H*=*μ*_0_*M*_s,p_≈1.6 T. However, the initial 1/*H* dependence in Fig. 3a clearly changes for fields well below *μ*_0_*M*_s,p_ where a local minimum is observed at about 0.8 T and the slope changes sign from negative to positive, suggesting that a different underlying mechanism starts to dominate.

## Discussion

To understand the change in the nucleation condition trend, we rely on the stability analysis proposed in ref. 17. For the experimental conditions reported above, the uniformly magnetized state is modulationally unstable and leads to the nucleation of a droplet. Consequently, it suffices to identify the current causing the onset of this instability. This is achieved by generalizing the Slonczewski critical current condition^35^ for a material with PMA and including perturbation terms proportional to the damping parameter. By solving an appropriate boundary value problem up to first order in *α* (see the ‘Methods' section for details), we obtain the nucleation current:


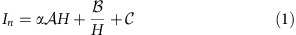


where 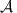, 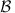 and 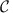 are given in the ‘Methods' section. In contrast to previously reported models^19^,^23^, a term proportional to *α* is observed, only arising from a first-order correction to the Slonczewski critical current condition. Moreover, as detailed in the ‘Methods' section, equation (1) includes the fixed layer hard axis reversal term 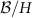, applicable when the applied field does not perpendicularly saturate the fixed layer and thus must depend on material parameters. The experimental data can be fitted very well by equation (1), as shown in Fig. 3a with solid lines following the same colour coding.

To test the validity of this model, we analyse the coefficients' dependence on the NC radius *R*_NC_, as shown in Fig. 3b,c. Both 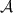 and 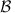 are found to depend on 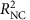, as predicted by the analytical model and the fits shown by the dashed lines. However, we are unable to find good agreement for the current shift 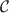. We argue that this disagreement stems from sample-to-sample variations and thermal excitation of magnons, which prevent the sharp spin wave onset. Indeed, the threshold current is typically one of the most variable parameters in NC-STOs and is usually estimated by projecting the thermally driven subthreshold power to zero^36^. This effect also has consequences on the zero-crossing of both 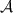 and 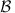 in the limit 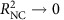, which returns negative values from our linear fits. Despite this disagreement, the slopes of 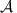 and 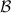 provide valuable information for determining intrinsic STT parameters, such as the spin-torque asymmetry (*λ*) and efficiency 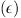. Utilizing the magnetic parameters specified above for our orthogonal NC-STO, we obtain *λ*=2.38±0.43 for the spin-torque asymmetry and 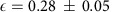 for the spin-torque efficiency, obtained from equation (24), equation (25) and equation (5) in the ‘Methods' section. The error of each parameter is obtained from 95% confidence bounds of the linear fits of 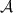 and 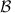 (Fig. 3a,b) and then applying error propagation.

Another characteristic of the nucleation current of equation (1) is the existence of a well-defined minimum, as observed experimentally. As noted above, the minimum occurs well below the field corresponding to a fully saturated fixed layer (*μ*_0_*M*_s,p_), indicating a more involved balance between the magnetic properties of the orthogonal NC-STO. By taking the derivative with respect to the field, we obtain a minimum current 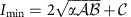 at 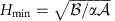. As for the calculation of the intrinsic STT parameters, we note that the slopes of 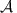 and 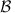 provide valuable information regarding the minimum current. Evaluating both *I*_min_ and *H*_min_, we obtain the proportionalities


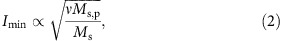



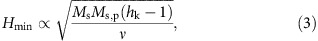


where *h*_k_=*H*_k_/*M*_s_ is the normalized anisotropy field and *ν*=(*λ*^2^−1)/(*λ*^2^+1) is the normalized asymmetry coefficient defined in the range 0≤*ν*<1. There are two interesting conclusions that we can draw from equations (2) and (3). On one hand, the minimum current from equation (2) indicates that a lower nucleation current can be achieved by a softer fixed layer and a harder free layer while striving for a small normalized asymmetry. On the other hand, the minimum field from equation (3) is minimized by softer free and fixed layers while it requires a nonzero normalized asymmetry to be defined. These relations suggest that the magnetic materials of the pseudo-spin-valve can be engineered to optimize the minimum current while balancing the operating field value. In addition, the existence of the current and field minimum is provided by a nonzero normalized asymmetry. In other words, the change in the nucleation condition trend at fields well below *μ*_0_*M*_s,p_ is direct proof of the asymmetric character of STT in metallic pseudo-spin-valves. Although theory predicts a substantial STT asymmetry in metallic pseudo-spin-valves, this has so far not been well reproduced experimentally in direct measurements, and the discrepancy has been ascribed to extrinsic device imperfections in the nanopillar geometry^37^. In the NC-STO geometry studied here, where the lateral extent of the spin valve removes any perimeter-related imperfections, droplet nucleation offers an alternative, straightforward and sensitive method for the accurate determination of the STT asymmetry, with *λ*=2.38±0.43 being much closer to theoretical predictions.

In conclusion, we have mapped out in detail the current–field nucleation conditions for droplets in orthogonal NC-STOs by using both microwave and resistance measurements. The observed nucleation boundary exhibits two different regimes in the limits of low and high fields. By extending the original droplet nucleation condition to include the tilt angle of the fixed layer and the spin transfer torque asymmetry, we are able to model our experimental results with excellent agreement. Our results hence directly reconcile the recent contradicting reports of the field dependence of droplet nucleation. An important consequence of this study is the existence of a current–field minimum for droplet nucleation, which can be tuned by the magnetic properties of the orthogonal NC-STO and, more strongly, by the spin-torque asymmetry of the fixed layer. These results are hence critical for the design of devices supporting droplets that aim to exploit their properties for applications with minimum field and current requirements, and also offer a direct and accurate method for detailed studies of the spin transfer torque asymmetry.

## Methods

### Sample fabrication

The orthogonal pseudo-spin-valves were deposited on thermally oxidized Si wafers using magnetron sputtering. The full stacks consisted of a pseudo-spin-valve with composition Co (6 nm)/Cu (6 nm)/Co (0.2 nm)[Ni (0.6 nm)/Co (0.25 nm)] × 4 with a Ta (4 nm)/Cu (10 nm)/Ta (4 nm) seed layer and a Cu (2 nm)/Pd (2 nm) cap layer. The wafer was patterned into a 8 × 16 μm^2^ mesa structure using optical lithography, and then coated with a 30 nm SiO_2_ interlayer dielectric deposited by chemical vapour deposition. The different NC sizes were fabricated using electron beam lithography and reactive ion etching through the SiO_2_. Finally, a 1.1 μm Cu top electrode was fabricated by optical lithography, sputter deposition and lift-off.

### Sample characterization

The fabricated orthogonal NC-STOs were characterized in our custom probe station, allowing us to control the field magnitude and angle independently. External fields up to 2 T can be generated by an electromagnet, while the angle is controlled by tilting the sample using a mechanical rotation between the out-of-plane direction (90^°^) and the in-plane direction (0^°^). Furthermore, both d.c. and microwave measurement can be performed simultaneously. The bias current is provided by a Keithley 6221 current source from which the MR can be monitored using a Keithley 2182A nanovoltmeter. The generated a.c. voltage is decoupled from the bias by using a d.c.-40 GHz bias-T. The a.c. signal is then amplified with a 0.1–30 GHz low-noise amplifier and analysed the frequency domain by a R&S FSU spectrum analyzer.

### Nucleation current derivation

The nucleation of droplets in NC-STOs coincides with Slonczewski's critical current condition^35^, including strong perpendicular anisotropy and weakly nonlinear effects^17^. The analytical expression for the nucleation current, equation (1), is derived by expressing the magnetization dynamics governed by the Landau–Lifshitz–Slonczewski equation as a function of the complex variable *u*=*m*_*x*_+*im*_*y*_ with the small amplitude approximation 
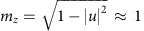
 in the form of a complex linear Schrödinger equation (see equation (30) in ref. 17)


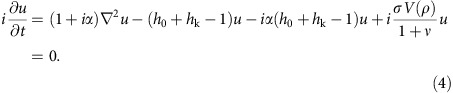


The parameters include the scaled applied field *h*_0_=*H*/*M*_s_, scaled anisotropy field *h*_k_=*H*_k_/*M*_s_ and nondimensionalized parameter of spin-transfer torque 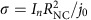, where the current density scaling is


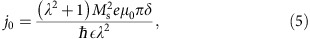


where *e* is the electron charge, *μ*_0_ is the vacuum permeability, *δ* is the thickness of the free layer, *ℏ* is the reduced Planck's constant, 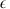 is the spin torque efficiency, 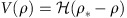 is a shifted Heaviside function and ρ_*_=*R*_NC_/*l*_ex_ is the scaled NC radius. Seeking a special solution in the form 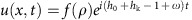, where *f*(*ρ*) is a radial function, ***x*** represents spatial coordinates, *ρ*=|***x***|, assuming 0<*α*<<1 and rescaling the spatial coordinate 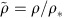 by the exchange length-scaled NC radius, we obtain the complex eigenvalue problem


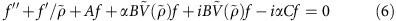


where


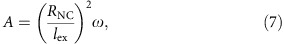



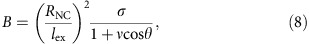



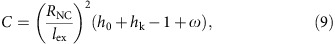



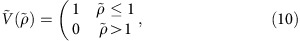


where *ω* is the precessional frequency at droplet onset and cos *θ*=*H*/*M*_s,p_ is the angle between the free and fixed layers, given by the hard axis reversal condition. Following refs 17, 35, we solve this by introducing the complex wavenumbers


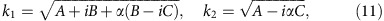


and a piecewise defined Bessel solution


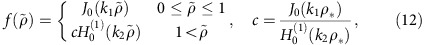


where *J*_0_ and 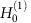 are the Bessel and Hankel functions of the first kind, respectively. To find the two real eigenvalues *ω* and *σ*, we require continuity of the derivative of *f* at the NC boundary 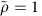, leading to the complex transcendental equation


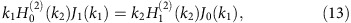


whose real and imaginary parts determine the eigenvalues. To simplify the calculation, we solve equation (13) by expanding the wavenumbers in the small parameter *α* as


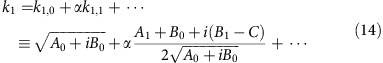



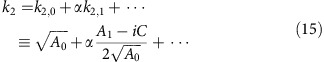


where we have also expanded the eigenvalue coefficients according to


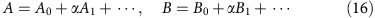


Note that *A*_0_ and *B*_0_ are simply *f*_0_ and *β*_0_ in ref. 35. The 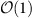 equation for equation (13) is


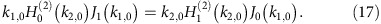


Solving equation (17) numerically gives


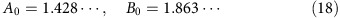


The 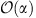 equation for equation (13) is


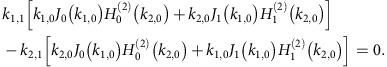


This is a linear equation for *A*_1_ and *B*_1_ whose solution is approximately


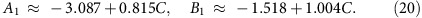


Using the definitions in equation (20) gives the approximate nucleation frequency and current for a dissipative droplet


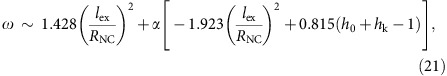



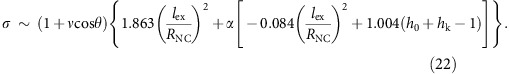


We are mainly interested in equation (22). We can express this equation in terms of current density via equation (5). By implementing this change and explicitly writing the hard axis reversal condition, we get


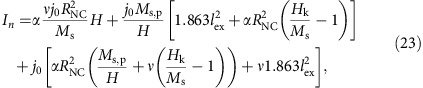


which is simply equation (1) by defining the coefficients


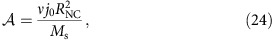



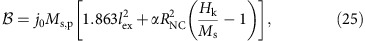



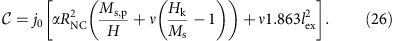


## Additional information

**How to cite this article:** Chung, S. *et al.* Magnetic droplet nucleation boundary in orthogonal spin-torque nano-oscillators. *Nat. Commun.* 7:11209 doi: 10.1038/ncomms11209 (2016).
