# Erratum: Magnetic droplet nucleation boundary in orthogonal spin-torque nano-oscillators

**DOI:** 10.1038/ncomms11699

**Published:** 2016-05-12

**Authors:** Sunjae Chung, Anders Eklund, Ezio Iacocca, Seyed Majid Mohseni, Sohrab R. Sani, Lake Bookman, Mark A. Hoefer, Randy K. Dumas, Johan Åkerman

Nature Communications 7 Article number: 11209 10.1038/ncomms11209(2016); Published: 04182016; Updated: 05122016.

The affiliation details for Mark A. Hoefer are incorrect in this Article. The correct affiliation details for this author are given below:

Department of Applied Mathematics, University of Colorado, Boulder, Colorado 80309-0526, USA.

